# Chronic Wasting Disease Prions in Elk Antler Velvet

**DOI:** 10.3201/eid1505.081458

**Published:** 2009-05

**Authors:** Rachel C. Angers, Tanya S. Seward, Dana Napier, Michael Green, Edward Hoover, Terry Spraker, Katherine O’Rourke, Aru Balachandran, Glenn C. Telling

**Affiliations:** University of Kentucky Medical Center, Lexington, Kentucky, USA (R.C. Angers, T.S. Seward, D. Napier, M. Green, G.C. Telling); Colorado State University, Fort Collins, Colorado, USA (E. Hoover, T. Spraker); US Department of Agriculture, Pullman, Washington, USA (K. O’Rourke); Canadian Food Inspection Agency, Ottawa, Ontario, Canada (A. Balachandran); 1 Current affiliation: Medical Research Council Laboratory of Molecular Biology, Cambridge, UK.

**Keywords:** Prions and related diseases, prion proteins, elk, antler velvet, chronic wasting disease, expedited, research

## Abstract

Chronic wasting disease (CWD) is a contagious, fatal prion disease of deer and elk that continues to emerge in new locations. To explore the means by which prions are transmitted with high efficiency among cervids, we examined prion infectivity in the apical skin layer covering the growing antler (antler velvet) by using CWD-susceptible transgenic mice and protein misfolding cyclic amplification. Our finding of prions in antler velvet of CWD-affected elk suggests that this tissue may play a role in disease transmission among cervids. Humans who consume antler velvet as a nutritional supplement are at risk for exposure to prions. The fact that CWD prion incubation times in transgenic mice expressing elk prion protein are consistently more rapid raises the possibility that residue 226, the sole primary structural difference between deer and elk prion protein, may be a major determinant of CWD pathogenesis.

## Abstract 2

Residue 226 of cervid prion proteins may be a determinant of CWD pathogenesis.

Chronic wasting disease (CWD) of deer, elk, and moose is the only recognized prion disease of wild animals. To date, 15 US states and 2 Canada provinces have reported CWD in wild and/or farm-raised cervids. Outbreaks have also occurred in South Korea as a result of importation of subclinically infected animals (*1*,*2*). The unparalleled efficiency of prion transmission in cervids by a largely undefined mechanism, combined with high deer densities in certain areas of North America, complicates strategies for controlling CWD as it continues to emerge in new locations.

Growing antlers of male cervids are covered by a highly innervated and vascularized apical skin layer, referred to as velvet, which is shed after an increase in testosterone and ossification of antlers. Our study objective was to assess whether velvet from CWD-infected elk contains prion infectivity. Our rationale was 2-fold. First, the annual shedding of this material raises the possibility that it may play a role in CWD transmission. Second, although the most likely means of human exposure to CWD prions is consumption of contaminated venison (*3*), the substantial market for velvet in traditional Asian medicine also warrants concern.

We used CWD-susceptible transgenic (Tg) mice as a sensitive means to detect prions in antler velvet. Bioassays in Tg mice expressing deer prion protein (PrP) (*4*) and newly created Tg mice expressing elk PrP, demonstrated low levels of CWD prions in antler velvet. We also show that the associated protease-resistant PrP could be amplified in vitro (for detection by Western blot) by protein misfolding cyclic amplification (PMCA). Finally, comparative CWD transmissions in Tg mice indicated that the glutamine (Q) to glutamic acid (E) variation at residue 226, which is the sole primary structural difference between deer and elk PrP, may be a major determinant of CWD pathogenesis in these 2 species.

## Materials and Methods

### Production of Transgenic Mice

Tg(CerPrP)1536^+/–^ mice expressing deer PrP have been described previously (*4*). To generate Tg(CerPrP-E226) mice expressing the elk PrP coding sequence, codon 226 of the deer PrP gene (*PRNP*) (GenBank accession no. AF009180) was mutated from Q to E by site-directed mutagenesis (Quick Change; Stratagene, La Jolla, CA, USA). The resulting expression cassette, CerPrP-E226, highlights the single amino acid difference between deer and elk PrP at this position (*5*). The coding sequence was inserted into the MoPrP.Xho expression vector, and the purified transgene was microinjected into pronuclei of fertilized FVB/*Prnp^0/0^* oocytes. Transgenic founders were identified by PCR screening of genomic DNA. Three Tg(CerPrP-E226) founders (Tg5029, Tg5034, Tg5037) were mated to FVB/*Prnp^0/0^* mice to produce hemizygous transgenic lines. Because of equivalent levels of CerPrP-E226 expression, studies were not duplicated in the Tg5034^+/–^ and Tg5037^+/–^ lines. Estimates of the levels of PrP expression in 3 different Tg5037^+/–^ mice and 5 different Tg5029^+/–^ mice were accomplished by immuno-dot blotting and Western blotting using monoclonal antibody (MAb) 6H4 (Prionics, Schlieren, Switzerland).

### Preparation of Prion Inocula

Antler velvet and matching brain samples were obtained from 4 elk from Canada that were naturally affected with CWD. Elk 01-0306 had severe CWD-specific neuropathologic changes in the obex and neurologic signs indicative of CWD; elk 02-0306 had moderate neuropathologic changes in the obex and was clinically normal. Although information about the clinical status of elk 03-0306 and 04-0306 was not available, these elk had mild and severe neuropathologic changes, respectively, in the obex. Brain samples were also obtained from CWD-affected mule deer D10 and D92 at the Colorado Division of Wildlife, Wildlife Research Center, and from CWD-affected elk 7378 and 99W12389 at the Wyoming Game and Fish Department’s Sybille Wildlife Research Unit. Homogenates of brain and antler velvet (10% wt/vol) were prepared in sterile phosphate-buffered saline (PBS) lacking Ca^2+^ and Mg^2+^ ions.

### Measurement of Incubation Times

Groups of 5-week-old Tg mice were given general anesthesia and inoculated with 30 µL of brain or antler velvet homogenate through a 27-gauge needle inserted into the right parietal lobe of the brain. Mice were observed 3 times a week for clinical signs indicative of prion infection, e.g., ataxia, weight loss, hyperactivity, flattened posture, absent extensor reflex, or kyphosis. The incubation period is the time between inoculation and the first day on which subsequently progressive clinical signs were identified. For end-point titration, groups of 8 Tg mice were inoculated with 10^–1^ to 10^–10^ dilutions of a 10% brain homogenate of D92 prepared in PBS lacking Ca^2+^ and Mg^2+^ ions.

### Western Blotting

Protein content in 10% brain homogenates was determined by bicinchoninic acid assay. Total protein (50 μg) was then digested with 40 μg/mL proteinase K (PK) (Roche, Mannheim, Germany) in the presence of 2% sarkosyl for 1 h at 37°C. Digestion was terminated with phenylmethylsulfonyl fluoride at a final concentration of 5 μM. Proteins were resolved by sodium dodecyl sulfate–polyacrylamide gel electrophoresis and transferred to polyvinylidene difluoride Immobilon-FL membranes (Millipore, Billerica, MA, USA), which were immunoprobed with MAb 6H4 followed by horseradish peroxidase–conjugated antimouse secondary antibody. Proteins were visualized by using ECL Plus (GE Healthcare, Piscataway, NJ, USA) and an FLA-5000 scanner (Fujifilm Life Science, Woodbridge, CT, USA).

### Histoblotting

Coronal cryostat sections (10 μm) were transferred to nitrocellulose and probed with MAb 6H4 after PK digestion as described previously (*6*). Images were photographed with a NikonDMX 1200F (Tokyo, Japan) digital camera in conjunction with Metamorph software (Molecular Devices, Sunnyvale, CA, USA).

### Immunohistochemical Analysis

Sections (8 μm) of formalin-fixed, paraffin-embedded mouse brains on positively charged microscope slides were deparaffinized and subjected to immunohistochemical analysis to deter the disease-associated form of PrP (PrP^Sc^) as described previously (*7*). PrP^Sc^ was detected with MAb 6H4 after hydrolytic autoclaving for 15 min in 10 mmol/L HCl. Biotinylated secondary antibody in conjunction with 3,3′-diaminobenzidine was used to visualize PrP^Sc^.

### PMCA

Healthy Tg(CerPrP)1536^+/–^ mice were perfused with PBS/5 mM EDTA. Brain homogenates (10% wt/vol) were prepared in conversion buffer consisting of PBS containing 150 mmol/L NaCl, 1.0% Triton X-100, and Roche’s Complete Protease Inhibitor Cocktail. Samples were clarified by centrifugation at 500 rpm for 60 s. Each PMCA cycle consisted of 30 min incubation at 37°C followed by a 20 s sonication pulse at setting 7 using a Misonix 3000 Sonicator (Misonix, Farmingdale, NY, USA). After 96 cycles, 6 μL of the 60-μL reaction was diluted into 54 μL of fresh Tg(CerPrP)1536^+/–^ substrate for a subsequent round of PMCA. Amplification products were digested with 50 μg/mL PK at 50°C for 75 min.

## Results

### CWD Prions in Elk Antler Velvet

Mean incubation periods for mice inoculated with CWD prions from elk brains were more uniform (225–335 days) than were those for mice inoculated with CWD prions from antler velvet. Not all Tg(CerPrP)1536^+/–^ mice inoculated with antler velvet developed disease; incubation times for those that did were relatively long and highly variable (Table). Clinical signs did develop in Tg(CerPrP)1536^+/–^ mice challenged with antler velvet from elk 01-0306 and 03-0306; mean incubation periods were ≈440 d and ≈460 d and attack rates were 75% and 66%, respectively. Tg(CerPrP)1536^+/–^ mice inoculated with antler velvet from elk 02-0306 and 04-0306 remained healthy until the mice were euthanized at ≈600 d postinoculation.

**Table:** Transmission of chronic wasting disease prions to transgenic mice*

Isolate from animal no.	Mice expressing deer PrP^C^			Mice expressing elk PrP^C^				
	Tg(CerPrP)1536^+/–^			Tg(CerPrP-E226)5037^+/–^			Tg(CerPrP-E226)5029^+/–^	
	Antler velvet	Brain		Antler velvet	Brain		Brain	% Decrease†
01-0306	442 ± 16 (6/8)	322 ± 9 (8/8)		305 ± 86 (2/7)‡	174 ± 7 (8/8)			46 (<0.0001)
02-0306	>594 (0/5)	225 ± 3 (7/7)						
03-0306	463 ± 23 (2/3)§	335 ± 5 (7/7)						
04-0306	>601 (0/6)	281 ± 5 (7/7)		>505 (0/5)	224 ± 6 (7/7)			20 (<0.0001)
7378		235 ± 2 (8/8)			177 ± 15 (7/7)			25 (0.0061)
99W12389		230 ± 9 (8/8)			158 ± 13 (7/7)			31 (0.0004)
D10		225 ± 1 (8/8)			201 ± 8 (8/8)			11 (0.013)
D92		268 ± 15 (7/7)			201 ± 15 (8/8)		263 ± 10 (7/7)	25 (0.0061)
PBS		>510 (0/6)						
None					>592 (0/4)¶			

*Incubation times are given as mean ± SEM days. Values in parentheses are no. diseased mice/no. inoculated mice. PrP^C^, cellular prion protein; Tg, transgenic; PBS, phosphate-buffered saline. †Values represent the percent decrease in mean incubation time of isolates in Tg(CerPrP-E226)5037^+/–^ mice compared with Tg(CerPrP)1536^+/–^ mice. p values, calculated by the Student *t*-test, are shown in parentheses. ‡The remaining 5 of 7 inoculated mice remained free of disease until 525 d postinoculation, at which time they were euthanized. §Mice inoculated with the antler velvet preparation from elk 03-0306 had an unusually high rate of intercurrent illnesses, resulting in early euthanasia of 5 inoculated mice. ¶Four uninoculated Tg(CerPrP E2226) 5037^+/–^ mice were euthanized at 437 d.

We also tested the susceptibility of Tg mice expressing elk PrP. Mean incubation times after inoculation of Tg(CerPrP-E226)5037^+/–^ mice with CWD prions from brains of elk 01-0306 and 04-0306 were 174 ± 7 d and 224 ± 6 d, respectively (Table). Of the Tg(CerPrP-E226)5037^+/–^ mice inoculated with antler velvet from elk 01-0306, 29% died of prion disease in <400 d, and antler velvet from elk 04-0306 failed to induce disease (Table); this finding confirmed the antler velvet transmission results in Tg(CerPrP)1536^+/–^ mice.

**Figure 1:**
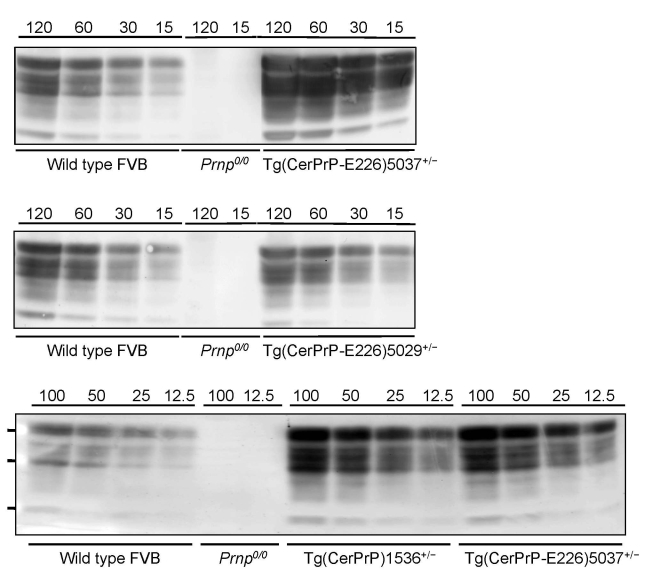
Levels of transgene expression in transgenic (Tg) mice expressing deer or elk cellular prion protein (PrP^C^). Representative Western blot analysis of PrP^C^ expression from different total protein loads in brain extracts from Tg mice Tg(CerPrP)1536^+/–^, Tg(CerPrP-E226)5029^+/–^, and Tg(CerPrP-E226)5037^+/–^ compared with wild type and *Prnp^0/0^* mice (knock-out mice for PrP gene).

Tg(CerPrP-E226)5037^+/–^ mice express PrP at ≈5-fold the level of PrP in the brains of wild type mice, similar to transgene expression levels in Tg(CerPrP)1536^+/–^ mice; the level of expression in Tg(CerPrP-E226)5029^+/–^ mice was approximately equal to that in wild type mice (Figure 1). Induction of disease by CWD prions in brain from diseased elk and deer was consistently and significantly more rapid in Tg(CerPrP-E226)5037^+/–^ than in Tg(CerPrP)1536^+/–^ mice (Table). Mean incubation times of the D92 isolate were equivalent in Tg(CerPrP)1536^+/–^ and Tg(CerPrP-E226)5029^+/–^ mice (Table), which have a 5-fold difference in transgene expression levels (Figure 1).

### PrP^Sc^ Accumulation and Neuropathologic Changes in Diseased Tg Mice

**Figure 2:**
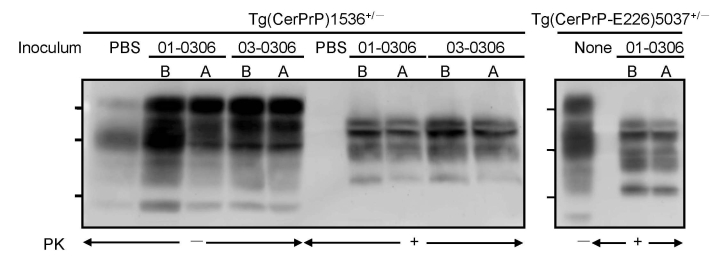
Accumulation of PrP^Sc^ (disease-associated form of prion protein) in diseased transgenic (Tg) mice. Tg(CerPrP)1536^+/–^ and Tg(CerPrPE226)5037^+/–^ mice inoculated with phosphate-buffered saline (PBS), elk brain (B), or antler velvet (A) were treated with or without proteinase K (PK). Membranes were probed with monoclonal antibody 6H4. Molecular weights indicated are 37, 29, and 20 kD.

**Figure 3:**
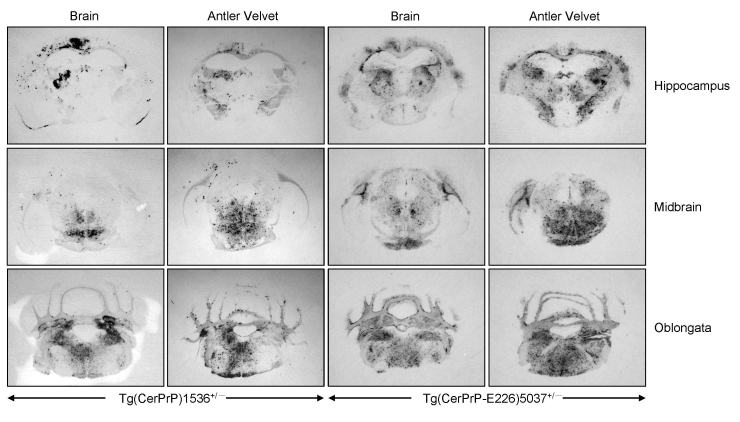
Distribution of PrP^Sc^ (disease-associated form of prion protein) in brains of diseased mice. Histoblots of mice inoculated with 01-0306 brain or antler velvet material were treated with proteinase K and probed with monoclonal antibody 6H4. Tg, transgenic.

**Figure 4:**
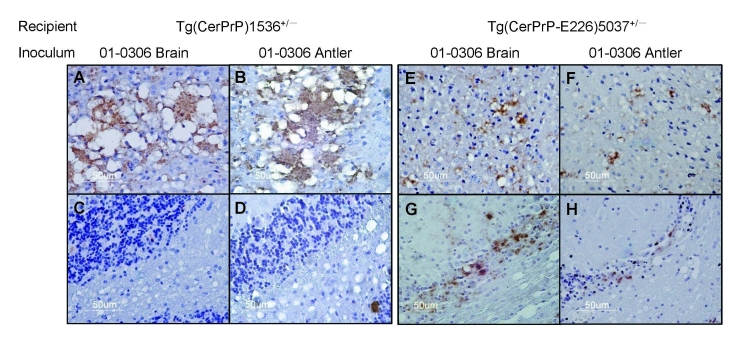
PrP^Sc^ (disease-associated form of prion protein)–specific immunohistochemistry in the brains of diseased mice. Transgenic (Tg) mice Tg(CerPrP)1536^+/–^ inoculated with brain (A) and antler velvet (B) preparations from elk 01-0306 exhibit florid PrP^Sc^-reactive plaques in the cerebral cortex at the level of the thalamus but retain integrity of cerebellar granular cells (C and D). Tg(CerPrP-E226)5037^+/–^ mice inoculated with brain (E) and antler velvet (F) preparations from elk 01-0306 display small plaques and diffuse granular staining in the cerebral cortex, PrP^Sc^ deposition, and marked cerebellar neuronal loss (G and H).

Diagnoses for all Tg(CerPrP)1536^+/–^ and Tg(CerPrP-E226)5037^+/–^ mice were confirmed by the presence or absence of protease-resistant PrP^Sc^ in brains, according to Western blotting (Figure 2) and histoblotting (Figure 3) and finding of disease-specific neuropathologic changes (Figure 4). PrP^Sc^ immunostaining in histoblots of diseased Tg(CerPrP)1536^+/–^ and Tg(CerPrP-E226)5037^+/–^ mice was punctate (Figure 3). Although cortical florid plaques were observed in the brains of diseased Tg(CerPrP)1536^+/–^ mice (Figure 4, panels A and B), PrP^Sc^ accumulation in Tg(CerPrP-E226)5037^+/–^ mice was more diffuse and granular. The extensive loss of cerebellar granular cells and accompanying PrP^Sc^ deposition that characterized disease in Tg(CerPrP-E226)5037^+/–^ mice (Figure 4, panels G and H) was not noted for Tg(CerPrP)1536^+/–^ mice (Figure 4, panels C and D).

### Estimates of CWD Prion Titers in Antler Velvet

**Figure 5:**
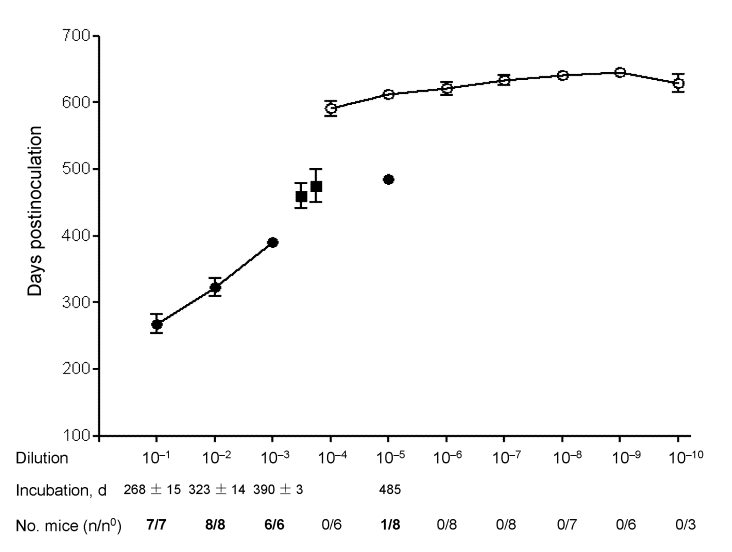
Quantification of chronic wasting disease prions. Diseased transgenic (Tg) mice Tg(CerPrP)1536^+/–^ inoculated with dilutions of brain homogenate are indicated by filled symbols Asymptomatic mice are indicated by open circles; time at which asymptomatic aged mice were either euthanized or died of illnesses unrelated to prion disease is shown. Error bars indicate SEM. n/n^0^ refers to the number of mice developing prion disease divided by the number of inoculated mice. Also shown are the mean incubation times of diseased Tg(CerPrP)1536^+/–^ mice inoculated with antler velvet preparations (filled squares).

A brainstem preparation from CWD-affected mule deer D92, which contained high levels of PrP^Sc^ according to Western blot (data not shown), was selected for end-point titration of CWD prions in Tg(CerPrP)1536^+/–^ mice. Disease developed in all mice inoculated with the 3 lowest dilutions; mean incubation periods ranged from 268 to 390 d (Figure 5). Disease did not develop in any of the mice inoculated with the 10^–4^ dilution, but disease did develop after 471 d in 1 mouse from the 10^–5^ dilution group. The remaining mice in the 10^–5^ dilution group and all mice inoculated with higher dilutions remained free of disease and were euthanized after 560–645 d. The disease status of all mice was confirmed by Western blotting (data not shown). We estimated the titer of CWD prions in D92 brain tissue to be 6 log i.c. ID_50_/g (i.c. ID_50_ refers to the dose of CWD prions that produces infection in 50% of the intracerebrally inoculated Tg mice) (*8*).

The inefficient transmission of prions from antler velvet samples (Table) is consistent with low levels of CWD prions. Because the incubation times of CWD prions in antler velvet from elk 01-0306 and 03-0306 were outside the linear range of dose and incubation time (Figure 5), we were unable to assign a definitive titer. The infection attack rates <100% suggested that CWD prion titers in these 1% inocula were close to, or at, the end point of the bioassay (<3.5 log i.c.ID_50_ units).

### Amplification of PrP^Sc^ in Antler Velvet Preparations

**Figure 6:**
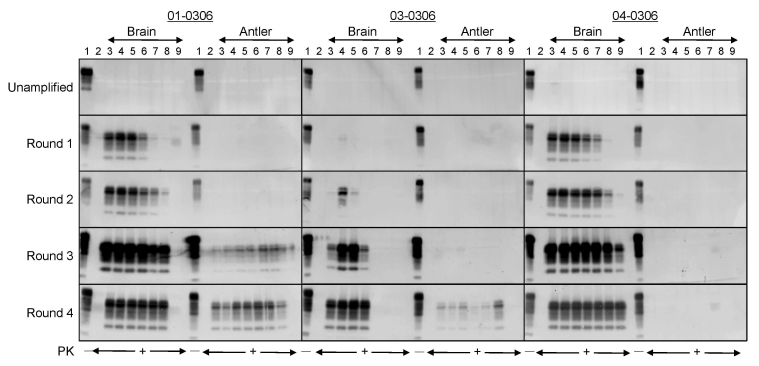
Detection of CerPrP^Sc^ (disease-associated form of cervid prion protein [PrP]) in brain and antler velvet from chronic wasting disease (CWD)–affected elk after serial protein misfolding cyclic amplification (PMCA). Western blots demonstrate amplification of protease-resistant prion protein (PrP) after serial PMCA when seeded with brain or velvet antler material from CWD-affected elk. Brain samples: lane 1, Tg(CerPrP)1536^+/–^ brain material not treated with proteinase K (PK); lane 2, Tg(CerPrP)1536^+/–^ brain material used as a negative control seed for the PMCA reactions; lanes 3–9, 10^–2^ to 10^–8^ dilutions of 10% elk brain homogenate. Antler velvet samples: lane 1, Tg(CerPrP)1536^+/–^ brain material not treated with PK; lane 2, Tg(CerPrP)1536^+/–^ brain material used as a negative control seed for the PMCA reactions; lanes 3–9, replicate 10^–2^ dilutions of 10% antler velvet homogenate. Samples were either treated or not treated with PK as indicated. Membranes were probed with monoclonal antibody 6H4. Tg, transgenic.

When Western blot, ELISA, and immunohistochemical analyses failed to detect PrP^Sc^ in antler velvet samples from 14 CWD-affected elk, including the 4 samples analyzed by bioassay (data not shown), we attempted to amplify PrP^Sc^ in antler velvet samples by using serial PMCA (Figure 6). Whereas PrP^Sc^ in brain homogenates of elk 01-0306, 03-0306, and 04-0306 was efficiently amplified, even at high dilution, after 1 or 2 rounds of PMCA, PrP^Sc^ in antler velvet homogenates of elk 01-0306 and 03-0306 was not amplified until either round 3 or 4. PrP^Sc^ was not amplified in antler velvet from elk 04-0306 or after PMCA of negative-control preparations. PrP^Sc^ amplification correlated with the transmission efficiency of CWD prions in antler velvet. The antler velvet sample from elk 01-0306, which produced detectable amplification products after 3 rounds of PMCA, caused disease in Tg(CerPrP)1536^+/–^ mice (mean incubation time 442 d, attack rate 75%) and in Tg(CerPrP-E226)5037^+/–^ mice. The velvet sample from elk 03-0306, which produced detectable amplification products after 4 rounds of PMCA, and resulted in a 463-d mean incubation time and a 66% attack rate. In contrast, the antler velvet sample from elk 04-0306, which failed to amplify after 4 rounds of PMCA, also failed to transmit prions to either Tg mouse line.

## Discussion

The transmission of CWD prions in antler velvet from 2 naturally affected elk to mice in 2 Tg models demonstrates that this tissue contains low, but detectable, amounts of CWD prions. In addition, serial PMCA amplified otherwise undetectable levels of PrP^Sc^ in antler velvet.

We characterized CWD prion infectivity by end-point titration. The ≈6 log i.c.ID_50_/g CWD prion titer estimated by this method contrasts with ≈9 log i.c.ID_50_/g titers of mouse-adapted scrapie prions in rodent brains (*9*) and ≈7–7.7 log i.c.ID_50_/g titers of BSE prions estimated by bioassay in transgenic mice (*10*,*11*). The linear relationship between dose and incubation time (*12*) provides an opportunity to estimate the level of prions in materials containing an unknown amount of infectivity. The attack rates of <100% after inoculation with antler velvet preparations from elk 01-0306 and 03-0306 and the failure to transmit disease from the remaining antler velvet samples suggest that CWD prion titers are close to, or at, the end point of the Tg(CerPrP)1536^+/–^ bioassay. Although we are aware of the limitations of comparing levels of prions in tissues from different CWD-affected cervids, we estimated the end point of the CWD prion titration using D92 to be <3.5 log i.c.ID_50_ units. Other factors could also influence levels of infectivity in the 4 tested samples, e.g., the portion of the antler processed and the age of the antler when harvested. Histologic evaluation indicated that the velvet samples used in these transmission studies came from elk antlers in the early stages of seasonal growth (data not shown). Whether CWD prion titers in antler velvet vary according to the state of antler growth remains to be determined. Whether prion infectivity is derived from nervous system tissue, blood (*13*), or another component of velvet, is also unclear.

### Implications for Horizontal CWD Transmission and Human Exposure

Our studies indicate that antler velvet represents a previously unrecognized source of CWD prions in the environment. Whereas oral transmission of rodent-adapted scrapie prions is known to be ≈5 orders of magnitude less efficient than transmission by intracerebral inoculation (*14*,*15*), the relative efficiency of oral CWD prion transmission is unknown. Multiple exposures to low levels of CWD prions in the environment (*16*,*17*), as well as increased infectivity when prions are bound to soil minerals (*18*), are factors that may influence transmission.

The appearance of variant Creutzfeldt-Jakob disease in humans exposed to bovine spongiform encephalopathy (BSE) (*19*,*20*) and the demonstration of CWD prions in muscle (*3*) placed the human species barrier to CWD prions at the forefront of public health concerns. Our studies indicate that antler velvet represents an additional source for human exposure to CWD prions. Widely used in traditional Asian medicine to treat a variety of ailments including impotence, arthritis, and high blood pressure, antler velvet can be readily purchased in caplet form and its usage has increased worldwide.

Fortunately, to date there is no epidemiologic evidence that rates of CJD in the CWD-endemic region (Colorado, USA) have increased (*21*,*22*). Also reassuring is the inefficient in vitro conversion of human PrP to protease-resistant PrP by CWD (*23*). Two studies have shown that CWD prions failed to induce disease in Tg mice expressing human PrP (*24*,*25*). However, the failure of BSE to be transmitted to Tg mice expressing human prion protein (HuPrP) was cited as early evidence of a BSE transmission barrier in humans (*26*); subsequent studies demonstrated a strong effect of the codon 129 polymorphism on transmissibility of BSE prions (*27*). To date, only mice expressing HuPrP with methionine at 129 have been challenged with CWD. In support of the argument that humans might be susceptible to CWD, intracerebral inoculation of squirrel monkeys produced disease after >30 months (*28*). Prion strain properties are also critical when considering the potential for interspecies transmission. The existence of multiple CWD strains has been suggested by several studies (*4*,*25*,*29*,*30*), but strain isolation and host range characterization have not been reported. Finally, it is worth considering that if CWD were to cross the species barrier into humans, this transmission source might not be recognized if the disease profile overlapped with one of the forms of sporadic CJD reported in North America.

### Possible Role for Residue 226 in CWD Pathogenesis

Previous studies that demonstrated more rapid CWD prion incubation times in Tg mice expressing elk PrP (*24*,*29*) than in Tg(CerPrP)1536^+/–^ mice (*4*) raised the possibility that the single amino acid difference at residue 226 between elk and deer PrP (*5*) may influence CWD pathogenesis (*29*). However, when the transmission characteristics of CWD isolates were directly compared in Tg mice expressing differing levels of deer or elk PrP, Tamgüney et al. concluded that CWD incubation times were related solely to the level of PrP transgene expression (*25*). We compared CWD transmission in Tg(CerPrP-E226)5037^+/–^ and Tg(CerPrP)1536^+/–^ mice, which express PrP at levels ≈5-fold higher than PrP levels in wild type mouse brain (Figure 1A), and found that CWD transmission was consistently and substantially more rapid in Tg(CerPrP-E226)5037^+/–^ mice. Our results appear compatible with more efficient CWD prion propagation by elk cellular prion protein (CerPrP^C^) containing E at residue 226 than by deer CerPrP^C^ containing Q at this position. Consistent with this interpretation, despite 5-fold lower levels of transgene expression in Tg(CerPrP-E226)5029^+/–^ mice than in Tg(CerPrP)1536^+/–^ mice, mean incubation times of the D92 isolate were equivalent in these 2 lines (Table). Nonetheless, undetected differences in CerPrP^C^ expression, for example in particular cell types, might result in more rapid disease and/or altered pathologic changes. The generation of transgenic mice expressing elk and deer coding sequences using gene replacement strategies would seem to be an excellent approach for resolving this issue.

The different responses to CWD in Tg mice also appear to recapitulate aspects of CWD pathogenesis in the natural hosts. Previous limited comparative transmission studies indicated that CWD developed ≈25% more rapidly in orally challenged elk than deer (*31*). Although plaques were not detected in brains of CWD-affected elk, florid plaques have been observed in the brains of diseased deer (*32*,*33*). Similar differences in pathologic changes were observed in Tg(CerPrP-E226)5037^+/–^ and Tg(CerPrP)1536^+/–^ mice (Figure 4). Structural analyses suggest that residue 226 is located within a region of PrP^C^ proposed to interact with a factor (*34*), possibly equivalent to the postulated protein X (*35*). Although mutation of the equivalent residue from Q to lysine (K) in epitope-tagged mouse PrP had no effect on PrP^Sc^ formation in transfected chronically infected ScN2A cells, the effects of the Q-to-E substitution were not assessed (*36*).
